# Sprouty1 is a broad mediator of cellular senescence

**DOI:** 10.1038/s41419-024-06689-4

**Published:** 2024-04-26

**Authors:** Carlos Anerillas, Aida Perramon-Güell, Gisela Altés, Sara Cuesta, Marta Vaquero, Anna Olomí, Ruth Rodríguez-Barrueco, David Llobet-Navàs, Joaquim Egea, Xavi Dolcet, Andrée Yeramian, Mario Encinas

**Affiliations:** 1 Developmental and Oncogenic Signaling Group, Universitat de Lleida/Institut de Recerca Biomèdica de Lleida, Rovira Roure, 80, Lleida, Spain; 2 Laboratory of Genetics and Genomics, National Institute on Aging, National Institutes of Health, 251 Bayview Blvd., Baltimore, MD USA; 3 Laboratory of Precision Medicine, Oncobell Program. Bellvitge Biomedical Research Institute (IDIBELL), Gran via De l’Hospitalet, Barcelona, Spain; 4 Present Address: Homeostasis de tejidos y órganos program, Centro de Biología Molecular Severo Ochoa, Consejo Superior de Investigaciones Científicas (CSIC) - Universidad Autónoma de Madrid, Madrid, Spain; 5 Present Address: Fundación de Investigación Biomédica de Cádiz, Hospital Universitario Puerta del Mar, Novena Planta, Investigación, Av Ana de Viya, 21, Cádiz, Spain; 6 Present Address: Hospital Universitari Arnau de Vilanova, Rovira Roure, 80, Lleida, Spain

**Keywords:** Cell biology, Molecular biology

## Abstract

Genes of the Sprouty family (Spry1-4) restrain signaling by certain receptor tyrosine kinases. Consequently, these genes participate in several developmental processes and function as tumor suppressors in adult life. Despite these important roles, the biology of this family of genes still remains obscure. Here we show that Sprouty proteins are general mediators of cellular senescence. Induction of cellular senescence by several triggers in vitro correlates with upregulation of Sprouty protein levels. More importantly, overexpression of Sprouty genes is sufficient to cause premature cellular senescence, via a conserved N-terminal tyrosine (Tyrosine 53 of Sprouty1). Accordingly, fibroblasts from knockin animals lacking that tyrosine escape replicative senescence. In vivo, heterozygous knockin mice display delayed induction of cellular senescence during cutaneous wound healing and upon chemotherapy-induced cellular senescence. Unlike other functions of this family of genes, induction of cellular senescence appears to be independent of activation of the ERK1/2 pathway. Instead, we show that Sprouty proteins induce cellular senescence upstream of the p38 pathway in these in vitro and in vivo paradigms.

## Introduction

Genes of the Sprouty family (Spry1-4) are feedback inhibitors of receptor tyrosine kinase (RTK) signaling conserved from flies to humans [1]. In mice, Spry genes mainly antagonize signaling by Ret and the FGF receptors (FGFRs). On the one hand, Spry1 inhibits Ret signaling during kidney morphogenesis [2, 3], whereas Spry2 does so during enteric nervous system development [4]. On the other hand, deletion of Spry2 and/or Spry4 causes different craniofacial abnormalities such as congenital deafness or diastema teeth owing to hypersensitivity to FGF [5, 6]. Likewise, Spry1 and Spry2 collaborate to shape the cerebellum, internal ear, eyes or internal genitalia downstream of the FGFRs [7–11].

How Spry proteins restrain RTK signaling is controversial. Several reports show that their main target is the ERK1/2 MAPK pathway [12], although the molecular mechanisms are less than clear [1, 13–15]. Gene targeting experiments from our laboratory demonstrate that a conserved N-terminal tyrosine (Tyrosine 53) of Spry1 is critical for its function, as knockin mice lacking this tyrosine phenocopy Spry1 knockout mice [16].

Due to its ability to restrain RTK signaling, Spry family members have been proposed to function as tumor suppressor genes. Accordingly, Spry levels are downregulated in tumoral vs normal human tissue. For example, Spry1 and Spry2 levels are decreased in prostate and breast cancer, whereas downregulation of Spry2 has been described in hepatocellular carcinoma, B-cell lymphoma, or endometrial carcinoma, among others [17]. More importantly, a causal relationship linking Spry deletion with cancer development has been established in prostate [18, 19] and thyroid [20], although deletion of Spry(s) alone is not sufficient for cancer development but requires concomitant Pten heterozygosity.

Cellular senescence is a state of proliferative blockade reached by either telomere attrition (replicative senescence) or by different sorts of cellular stresses (stress-induced senescence) such as oxidative stress, DNA damage, or oncogene expression (oncogene-induced senescence, OIS) [21–23]. Senescent cells are characterized by the expression of cell cycle inhibitors such as p16^Ink4a^, and/or by activation of the p53/p21^Cip1^ axis. Besides expression of these cell cycle inhibitors, senescent cells secrete a complex cocktail of molecules collectively known as the senescence-associated secretory phenotype (SASP). Finally, senescent cells are usually identified by their ability to metabolize the chromogenic substrate X-gal at acidic pH (senescence-associated β-galactosidase activity or SA-β-Gal), although this feature is not exclusive of senescent cells.

Cellular senescence plays beneficial roles in processes such as tissue remodeling and tumor suppression but paradoxically contributes to aging and aged-related diseases, thus constituting a double-edged sword to organismal homeostasis [22]. Recently, a novel form of cellular senescence that helps patterning the embryo during development has been described [24, 25]. Programmed cellular senescence shapes the embryo by remodeling or eliminating embryonic structures such as the endolymphatic sac, the ectodermal apical ridge, the interdigital membranes, the mesonephric tubules, or the Wolffian duct of female mice [23].

Our previous work suggests that Spry genes, and more specifically Spry1, are inducers of cellular senescence. First, overexpression of Spry1 in a thyroid cancer cell line reduces its proliferation in vitro and in xenografts, concomitant with an increase of p16^ink4a^ and SA-β -Gal [26]. Second, Spry1 knockout mice exhibit overgrowth of the thyroid gland in vivo, accompanied by decreased SA-β-Gal and SASP factors [20]. Finally, caudal Wolffian Ducts from heterozygous Spry1^Y53A/+^ females fail to degenerate but remain to adulthood, a phenotype that is consistent with defective implementation of programmed cellular senescence [7]. These observations led us to investigate whether Sprouty proteins function as broad mediators of cellular senescence.

In this work, we present evidence that Sprouty proteins are critical mediators of cellular senescence. Exposure of human IMR90 fibroblasts to different cellular senescence triggers increase expression of Sprouty proteins. Moreover, overexpression of Spry1-4 induces cellular senescence of IMR90 cells, via a mechanism that depends on the conserved N-terminal tyrosine. Accordingly, skin fibroblasts from Spry1^Y53A/Y53A^ knockin mice escape cellular senescence in vitro, and heterozygous Spry^Y53A/+^ mice display reduced wound healing and doxorubicin-mediated cellular senescence responses. Mechanistically, we show that Sprouty-induced senescence is independent of FGF2 or ERK signaling but occurs upstream of the p38 pathway both in vivo and in vitro.

## Results

### Sprouty family members are upregulated upon induction of cellular senescence

**Fig. 1:**
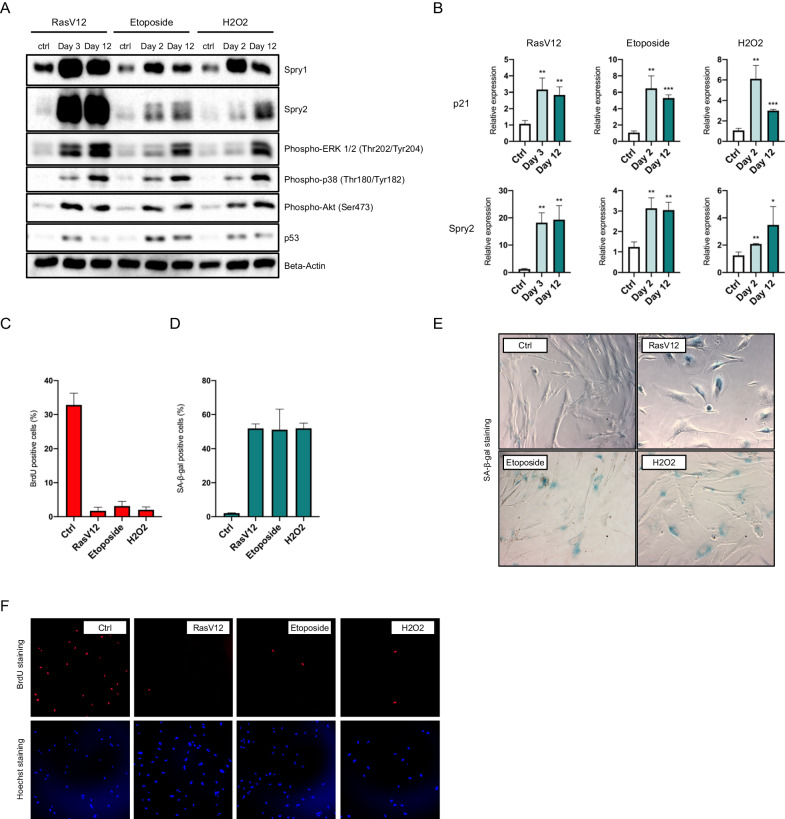
Sprouty proteins are upregulated upon induction of cellular senescence. **A** Protein levels of Sprouty1 and Sprouty2 were analyzed at the indicated time points after triggering cellular senescence with RasV12, Etoposide, or H_2_O_2_. Additionally, different signaling pathways associated with cellular senescence dynamics were analyzed. Results are representative of three independent experiments. **B** RT-qPCR analysis of the expression of p21 and Spry2 transcripts at the indicated time points after senescence induction (*n* = 3 samples per time point). **C** Quantification of 5-bromo-2’-deoxyuridine (BrdU) and **D** SA-β-Gal positive cells at day 12 after senescence induction (*n* = 2 per condition). **E**, **F** Representative images of SA-β-Gal (**E**) or BrdU (**F**) staining performed for each of the senescence triggers used at day 12. For all graphs, values represent mean ± SD. Student’s t-test was used for all comparisons among conditions with at least *n* = 3 replicates (*P < 0.05, **P < 0.01, ***P < 0.001).

As mentioned above, a series of previous observations from our laboratory suggested that Sprouty family members could be critical for implementation of a cellular senescence response. To explore that hypothesis we first confirmed whether Sprouty proteins become upregulated upon induction of cellular senescence. To do so, IMR90 fibroblasts were exposed to different senescence triggers such as oncogenic Ras, the DNA damaging drug etoposide and the oxidative stress agent hydrogen peroxide. As shown in Fig. 1A, all three treatments produced robust and long-lasting increases of both Spry1 and Spry2 protein levels, together with chronic activation of the ERK1/2, p38, and Akt signaling pathways. This activation was not due to increases in the steady state levels of ERK1/2, p38, or Akt proteins (Supplemental Fig. 1). Analysis of Spry2 mRNA levels indicated that such response was at least in part transcriptional (Fig. 1B). Correct implementation of cellular senescence was confirmed by induction of p53 (Fig. 1A), expression of the cell cycle inhibitor p21^CIP1^ (Fig. 1B), decrease in 5’-bromo-deoxyuridine uptake (BrdU, Fig. 1C, F), and positive staining for senescence-associated β-galactosidase activity (SA-β-Gal, Fig. 1D, E).

### Sprouty family members are necessary and sufficient for induction of cellular senescence

**Fig. 2:**
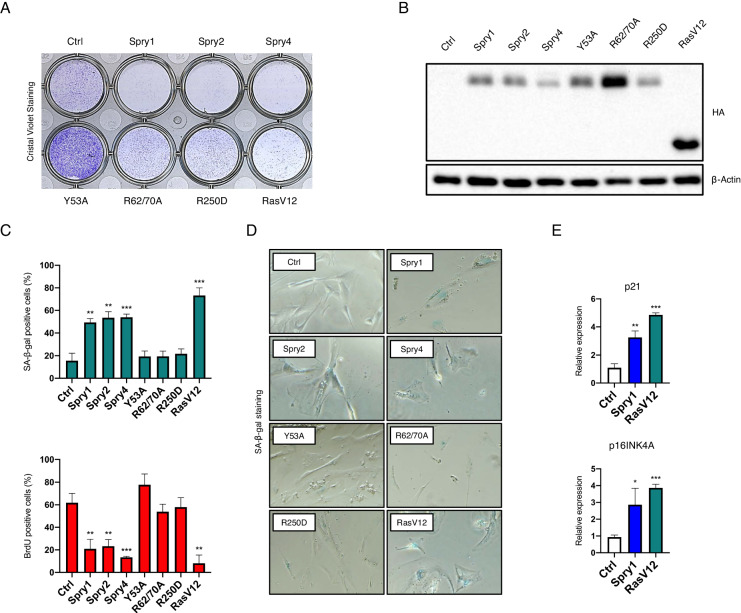
Sprouty proteins are sufficient to induce cellular senescence. **A** Representative image of clonogenic assays of IMR90 fibroblasts expressing the indicated constructs. **B** Immunoblot showing proper expression of all HA-tagged constructs used in these experiments. **C** Quantification of SA-β-Gal (blue graph) and 5-bromo-2’-deoxyuridine (red graph) staining at day 21 (Sprouty) or at day 12 (RasV12) post-infection from *n* = 3 independent experiments. **D** Representative images of SA-β-Gal staining at the same time points. **E** RT-qPCR of p21 and p16INK4A transcripts normalized to ACTB expression at the same time points. Shown are mean ± SD of *n* = 3 independent experiments. *P < 0.05, **P < 0.01, ***P < 0.001 by two-tailed Student’s t-test.

We next investigated the biological relevance of the above rise of Sprouty protein levels in response to cellular senescence inducers. We first tested whether expression of Sprouty family members was sufficient to induce cellular senescence of IMR90 cells. As shown in Fig. 2A, colony formation assays demonstrated that lentiviral-driven expression of all three Spry1, Spry2, and Spry4 caused a reduction of cell number in crystal violet-stained cultures dishes. Since all these proteins were HA-tagged, we confirmed their expression by means immunobloting against HA (Fig. 2B). SA-β-Gal and BrdU staining (Fig. 2C, D), as well as expression levels of mRNA coding for p21^CIP1^ and p16^INK4a^ (Fig. 2E) confirmed that reduction in cell numbers was due to induction of cellular senescence. Expression of oncogenic Ras (RasV12) served as positive control.

We next generated a series of point mutants of Spry1 in order to ascertain which residue(s) could be important to mediate induction of cellular senescence. First, we included the Y53A mutant as this tyrosine has been shown to be critical for Sprouty1 function during genitourinary development [16]. We also tested a double Arginine to Alanine (R62/70A) mutant lacking essential residues for biding of Sprouty to CIN85, an effector of NFκB signaling in B-cells [27]. Single mutants of these two Arginines did not show any effect on cellular senescence (Supplementary Fig. 1). We finally included the R250D mutant, which is defective for binding to Caveolin-1, a relevant interactor of Spry proteins [28], previously linked to cellular senescence [29]. Other candidate residues found in human cancer were analyzed but showed no effect of cellular senescence induction (Supplementary Fig. 1). As shown in Fig. 2A, mutation R62/70 A and R250D prevented Spry1-induced cellular senescence, while mutation of Tyrosine 53 showed colony densities well above those attained by control (vector-infected) IMR90 fibroblasts, in accordance with the dominant-negative nature of that mutation both in vivo and in vitro [7, 30]. Again, SA-β-Gal and BrdU staining confirmed that mutation of these residues effectively prevented implementation of cellular senescence (Fig. 2C, D).

**Fig. 3:**
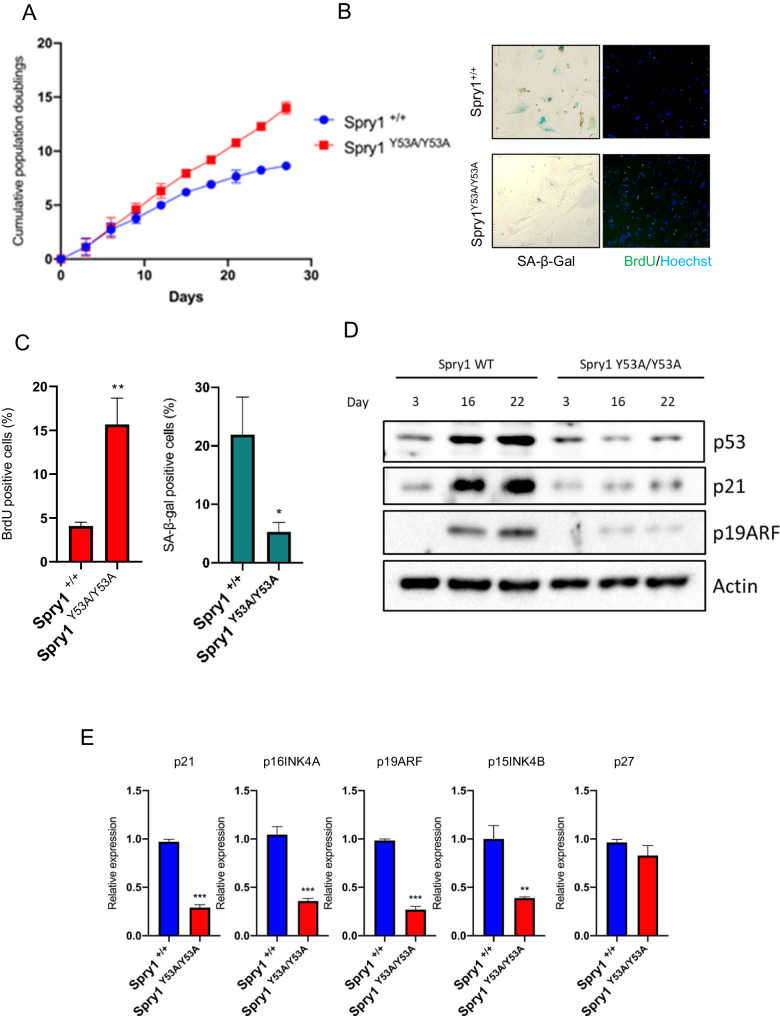
Sprouty1 induces cellular senescence via its N-terminal tyrosine in vitro. **A** Population doublings of newborn skin fibroblasts from mice of the indicated genotypes cultured under a 3T3 scheme. Values represent mean ± SD of independent cultures from six Spry1^+/+^ and four Spry1^Y53A/Y53A^ pups. **B** Representative pictures of SA-β-Gal and 5-bromo-2’-deoxyuridine (BrdU) staining of skin fibroblasts from the indicated genotypes at day 22. **C** Quantification of BrdU (red graph) and SA-β-Gal (blue graph) after 22 days in culture. **D** Representative panel of a time course assessment of cellular senescence markers by Western Blot at the indicated times for the stated genotypes. **E** Messenger RNA levels of the indicated cell cycle inhibitors at day 22 in fibroblasts from Spry1^+/+^ and Spry1^Y53A/Y53A^ mice (*n* = 3). Data were normalized to ACTB expression of cells from one wild type animal at day 22. *P < 0.05, **P < 0.01, ***P < 0.001 by two-tailed Student’s t-test.

These observations indicate that overexpression of Sprouty proteins is sufficient to promote cellular senescence, with a major contribution of Tyrosine 53 to the process. To ascertain whether Sprouty proteins are necessary for senescence implementation, we isolated skin fibroblasts from Spry1^Y53A/Y53A^ mutant mice, placed it in culture in a 3T3 scheme, and compared them to wild-type littermates. As shown in Fig. 3A, after around 15 days in vitro population doublings of wild-type fibroblasts reached a plateau whereas fibroblasts from Spry1^Y53A/Y53A^ knockin mice kept proliferating at a constant rate until the last time point analyzed. Examination of BrdU uptake and SA-β-Gal staining at day 22 confirmed that reduction in proliferative potential of wild-type skin fibroblasts was due to induction of cellular senescence (Fig. 3B, C). To further characterize the senescence process of these fibroblasts, we examined protein levels of p53 and cell cycle inhibitors p21^Cip1^ and p19^Arf^, as well as mRNA levels of these cell cycle inhibitors plus those of p16^Ink4a^, p15^Ink4b^, and p27^Kip1^ (Fig. 3D, E). In all cases except for p27^Kip1^, wild-type fibroblasts displayed higher levels of these senescence mediators.

### Sprouty1 is a critical mediator of cellular senescence in vivo

**Fig. 4:**
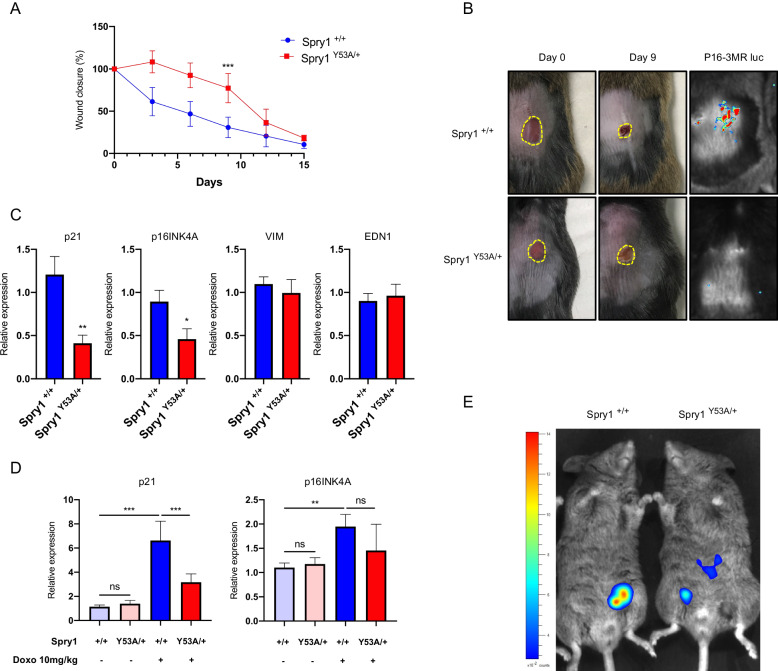
Mutation of the N-terminal tyrosine of Spry1 attenuates cellular senescence in vivo. **A** Dynamics of skin wound closure calculated as the percentage of initial wound area of *n* = 7 male mice of the indicated genotypes. **B** Left and middle columns, representative images of cutaneous wounds of wild type or Spry1^Y53A/+^ mice at day 0 and 9 days later. Right columns, luciferase activity of Spry1^+/+^; p16-3MR or Spry1^Y53A/+^; p16-3MR mice at day 9 after wounding, where cellular senescence peaks in males. **C** Expression levels of the specified genes in wound biopsies of the indicated genotypes (*n* = 3 each). **D** Expression levels in lungs from Doxorubicin-treated mice of the indicated genotypes [untreated Spry1^+/+^ (*n* = 3), untreated Spry1^Y53A/+^ (*n* = 3), Doxo-treated Spry1^+/+^ (*n* = 6), and Doxo-treated Spry1^Y53A/+^ (*n* = 6)]. **E** Lucíferase activity of Spry1^+/+^‘; p16-3MR or Spry1^Y53A/+^; p16-3MR mice 10 days after doxorubicin injection. *P < 0.05, **P < 0.01, ***P < 0.001 by two-tailed Student’s test.

The above experiments confirmed that Sprouty proteins are crucial for execution of cellular senescence in vitro. We next wanted to test whether the same was true in vivo. To that end, we used Spry^Y53A/+^ heterozygous mice to avoid complications derived from the kidney genotype of homozygous knockin mice. We used two in vivo paradigms in which induction of cellular senescence is relevant, namely wound healing [31] and chemotherapy-induced senescence [32]. In the first case, cutaneous wounds were made in Spry1^Y53A/+^ and wild type mice and the time course of healing was followed by measuring the wound area over time. As shown in Fig. 4A, a delay in wound closure consistent with suboptimal cellular senescence implementation was observed in Spry1 heterozygous knockin mice. Since wound closure can be affected by various processes besides cellular senescence such as migration or angiogenesis, we sought to ascertain whether the observed effects could be attributed to defective senescence in Spry1^Y53A/+^ mice. On the one hand, we crossed these mice to transgenic mice expressing Renilla luciferase under the transcriptional control of the p16 locus and thus reporting cellular senescence [p16-3MR [31]]. Bioluminescence assays showed luciferase activity in wounds from wild type but not Spry1^Y53A/+^ mice (Fig. 4B). On the other hand, analysis of mRNA expression of p21^Cip1^ and p16^Ink4a^ demonstrated a decrease of these cellular senescence markers in wounds (Fig. 4C). Importantly, Vimentin and Endothelin1 expression was similar in wounds from mice of the two genotypes, indicating that such decrease was not due to reduced numbers of fibroblasts or endothelial cells, respectively.

For chemotherapy-induced senescence, we injected Spry1^+/+^ or Spry1^Y53A/+^ mice bearing the p16-3MR transgene with a single dose of the chemotherapeutic drug doxorubicin, a common trigger of cellular senescence [31, 33, 34]. As shown in Fig. 4D, doxorubicin-induced expression of p21^Cip1^ in lungs was reduced in mutant mice, consistent with defective senescence responses in these animals. On the other hand. expression of p16^ink4a^ was not significantly reduced in Spry1^Y53A/+^ mice, likely due to the modest induction of its mRNA in this experimental paradigm. Alternatively, unlike p21-positive cells, deletion of a single allele of Spry1 might not be enough to cause a significant reduction of the number of p16-positive senescent cells in this experimental paradigm. Finally, a reduction of Renilla luciferase bioluminescence indicative of reduced cellular senescence was observed in Spry^Y53A/+^ mice (Fig. 4E).

### Inhibition of ERK or FGF signaling does not underlie Spry1-induced cellular senescence

**Fig. 5:**
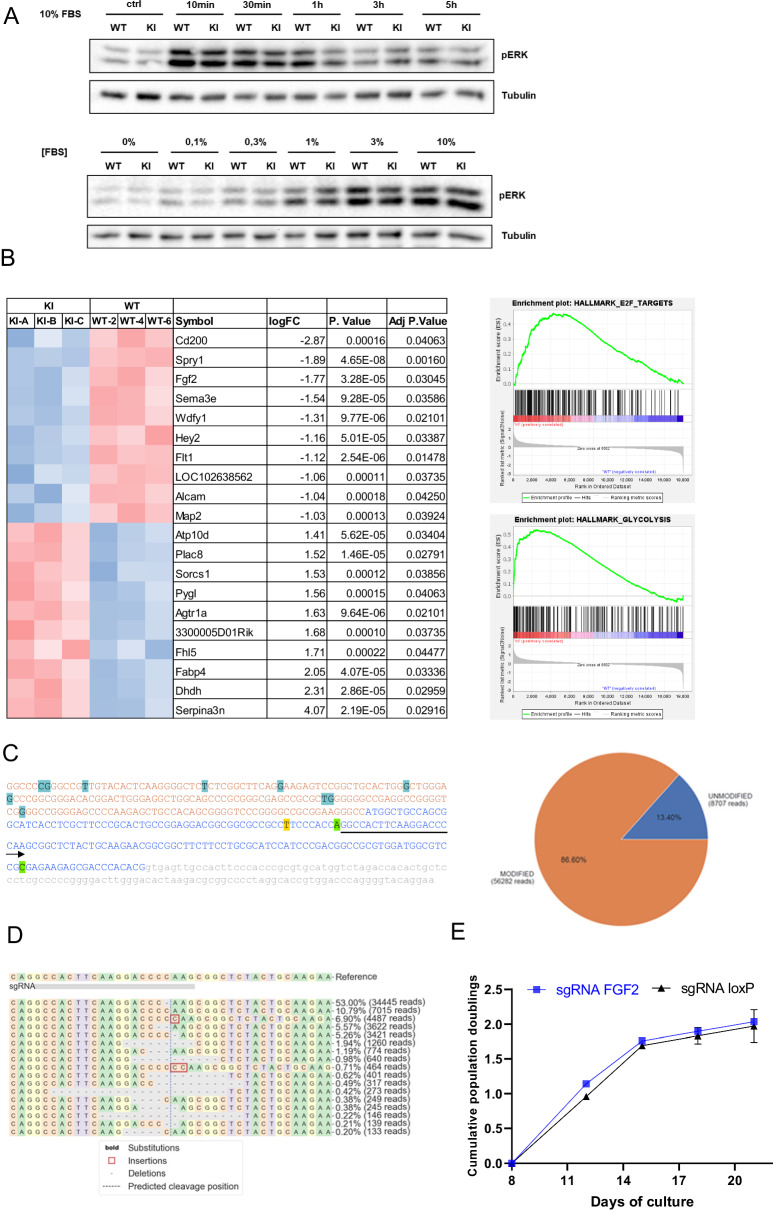
Sprouty-induced cellular senescence is independent of Fgf2 and the ERK pathway in dermal fibroblasts. **A** Skin fibroblasts from the indicated genotypes were deprived from FBS for 3 h and stimulated by the addition of FBS-containing media at the stated concentrations or for the indicated times. **B** Transcriptomic analysis shows downregulation of Fgf2 and upregulation of E2F targets and glycolytic genes in knockin skin fibroblasts. **C** Left panel, sequence of the first exon of the mouse Fgf2 gene, with arrow depicting the sgRNA sequence (Orange, 5’-UTR; blue, coding sequence containing ATG; gray, intronic sequence). Right panel, efficiency of gene editing was close to 90%. **D** Frequency of most common indels disrupting the Fgf2 gene generated by CRISPR/Cas9. **E** Edited fibroblasts expressing the sgRNA targeting Fgf2 enter cellular senescence with the same kinetics than those bearing a control sgRNA targeting loxP.

The ERK1/2 MAPK pathway is considered to be the main target of Sprouty family members. We therefore examined whether the effects of Y53A mutation on senescence of dermal fibroblasts could be caused by hyperactivation of the ERK pathway. To do so, we stimulated cells with FBS and measured ERK phosphorylation as a readout of pathway activation. As shown in Fig. 5A, the time course and dose-dependency of FBS-meditated ERK1/2 phosphorylation was not affected by mutation of Spry1 Tyrosine 53, suggesting that such pathway does not play a role in Sprouty-induced cellular senescence. To gain insight into the molecular mechanisms downstream of Sprouty-mediated senescence, we conducted a transcriptomic analysis of dermal fibroblasts from wild type and Y53A knockin mice after two weeks in culture. We chose this time point to detect gene expression changes preceding cellular senescence. GSEA analysis of expression patterns revealed increases in E2F targets and genes of the glycolytic pathway in knockin fibroblasts, consistent with wild type but not mutant cells entering a pre-senescent state [Fig. 5B [35]]. Interestingly, Fgf2 was among the top-ten downregulated genes in knockin fibroblasts (Fig. 5B). Given the role of Sprouty genes as inhibitors of FGF signaling, this could be interpreted as a cellular response to excessive signaling by the FGF receptors, perhaps independently of the ERK1/2 pathway. To assess the effect of eliminating Fgf2 expression in this paradigm we edited that gene in wild-type fibroblasts via CRISPR/Cas9 and analyzed induction of cellular senescence by serial passaging. Sequencing of the Fgf2 locus around the Cas9 cleaving site revealed that close to 90% of the sequences were edited (Fig. 5C). More than 50% of the reads corresponded to a single nucleotide deletion causing a frameshift mutation giving rise to a truncated protein retaining only the first 28 aminoacids of FGF2 protein (Fig. 5D). Other, less abundant indels also caused frameshift mutations (Fig. 5D). On the contrary, only 0.5% of reads from cells expressing a control sgRNA targeting loxP sequences were modified around the Fgf2 sgRNA cleavage site, probably representing sequencing errors (Supplemental Fig. 2). However, as shown in Fig. 5E, cells expressing the sgRNA to Fgf2 entered cellular senescence with the same kinetics as those expressing the control loxP sgRNA, indicating that inhibition of FGF2 signaling does not underlie Spry1-induced cellular senescence in this experimental paradigm.

### Sprouty family members induce cellular senescence upstream of the p38 pathway

**Fig. 6:**
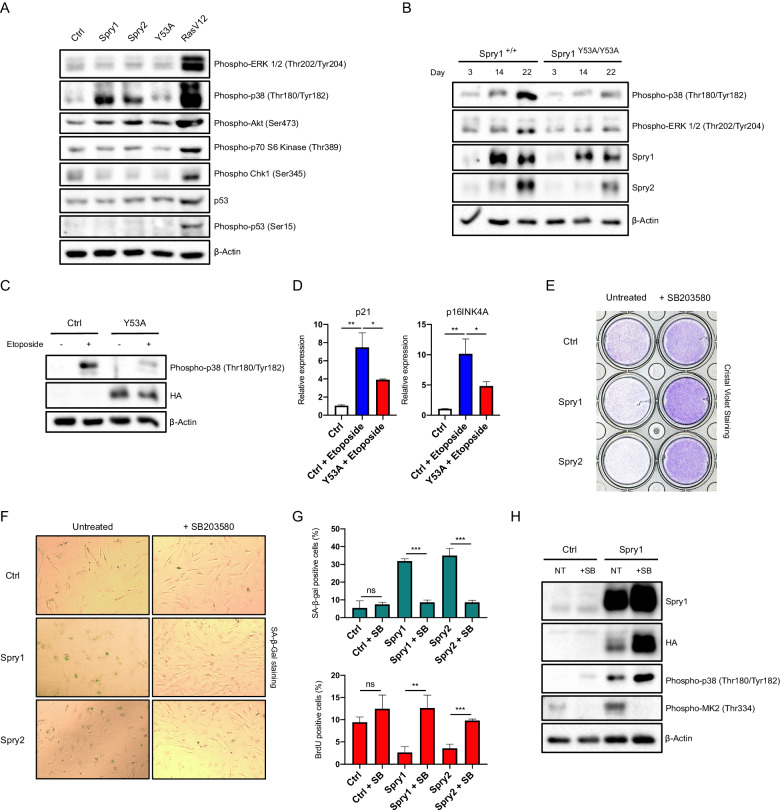
Sprouty proteins induce senescence through a p38-dependent mechanism. **A** Immunoblots showing robust activation of the p38 pathway among several senescence-related pathways analyzed in IMR90 cells overexpressing the indicated lentiviral constructs, 22 days after infection. **B** Activation of the ERK1/2 and p38 pathways and protein levels of Spry1 and Spry2 over time in culture of skin fibroblasts of the indicated genotypes. **C** Representative immunoblots of IMR90 cells infected with empty vector or HA-tagged Spry1 Y53A, 12 days after Etoposide treatment. **D** RT-qPCRs of p21 and p16INK4A transcripts measured for the indicated experimental conditions in IMR90 cells (*n* = 3 each group). **E** Representative clonogenic assays of IMR90 expressing the indicated constructs and continuously treated or not with SB203580. **F** Representative pictures of SA-β-Gal staining of IMR90 expressing the indicated constructs and treated or not with SB203580. **G** Quantification of SA-β-Gal- or BrdU-positive cells for each of the groups indicated (*n* = 3 each condition). **H** Representative Western Blot of empty vector- or Spry1-overexpressing IMR90 cells 21 days after being infected with each lentiviral construct. Values are mean ± SD. *P < 0.05, **P < 0.01, ***P < 0.001 by two-tailed, Student’s t test.

We next wanted to explore alternative mechanisms by which Sprouty proteins could modulate cellular senescence. We first examined ERK1/2 phosphorylation of IMR90 fibroblasts expressing Spry1-2 and of skin fibroblasts from wild-type mice and Spry1^Y53A/+^ littermates over time in culture to rule out chronic ERK inhibition as a mediator of Sprouty-induced senescence. As shown in Fig. 6A, overexpression of Sprouty proteins in IMR90 cells did not have a chronic effect on activation of the ERK1/2 MAPK pathway as measured by ERK1/2 phosphorylation. Other signaling pathways such as the PI3-K/Akt, were also unaffected by overexpression of Sprouty proteins. DNA damage-sensing pathways were inactive too as judged by absent Chk1 and p53 phosphorylation. Oncogenic Ras expression served as a positive control (Fig. 6A). Besides ERK1/2, p38 is the MAPK most commonly linked to senescence [36]. Remarkably, phosphorylation of p38 was prominent in Spry1- and Spry2-overexpressing cells, but not in vector-infected or Spry1Y53A-expressing IMR90 fibroblasts. Accordingly, skin fibroblasts from Spry1^+/+^ mice showed a time-dependent increase in p38 phosphorylation that was greatly attenuated in cells from Spry1^Y53A/Y53A^ mice (Fig. 6B). Likewise, etoposide exposure prompted a chronic induction of the p38 pathway that was virtually impaired by expression of Spry1 Y53A mutant in IMR90 fibroblasts (Fig. 6C). Consequently, levels of p21^Cip1^ and p16^Ink4a^ transcripts were defectively induced in Y53A-overexpressing IMR90 cells after full induction of senescence with Etoposide (Fig. 6D).

To test whether chronic activation of the p38 pathway was instrumental in induction and/or maintenance of cellular senescence in our models, we incubated cells with SB203580, a selective inhibitor of p38, and measured induction of cellular senescence in IMR90 fibroblasts overexpressing Spry1 or Spry2. Long-term inhibition of the p38 pathway prevented Sprouty-induced implementation of cellular senescence, as judged by colony formation assays (Fig. 6E), as well as SA-β-Gal staining and BrdU uptake (Fig. 6F, G). To rule out the possibility that the observed effects were due to downregulation of Sprouty proteins by SB203580, we examined levels of Sprouty1 in the presence and the absence of the drug. As shown in Fig. 6H, blockade of the p38 pathway by SB203580 did not result in decreased levels of Sprouty1, but rather caused its accumulation. Phosphorylation of the p38 target MAKAP kinase-2 (MK2) confirmed chronic inhibition of the p38 pathway (Fig. 6H).

**Fig. 7:**
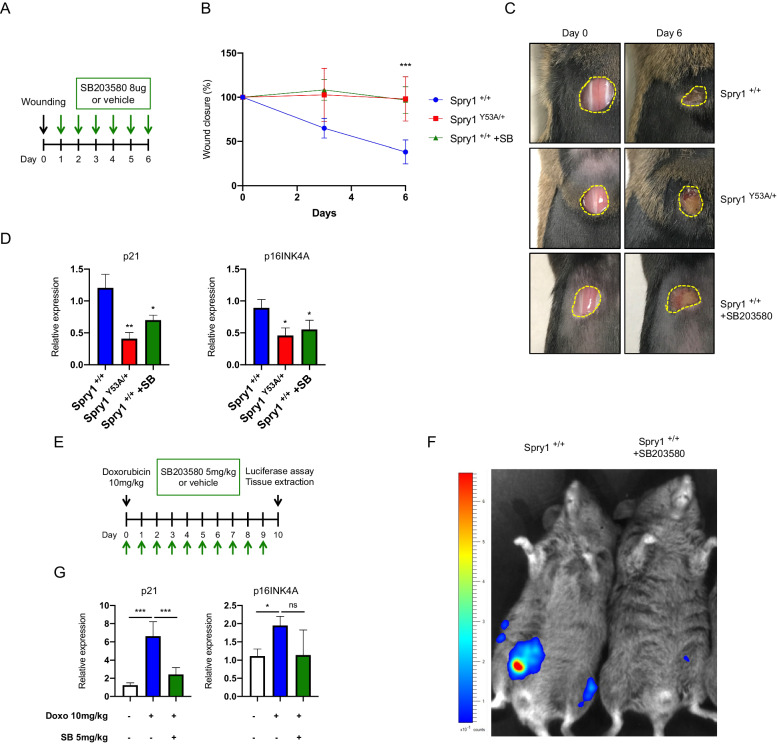
Wound closure and Doxorubicin-induced cellular senescence are dependent on p38 activity. **A** Experimental design and **B** wound closure kinetics of mice of the following genotypes: Spry1^+/+^ (*n* = 5), Spry1^Y53A/+^ (*n* = 2), Spry1^+/+^ treated with SB203580 (*n* = 5). For this series of experiments, female mice were used as they show accelerated wound closure, with cellular senescence in damaged skins peaking at day 6. **C** Representative images of the wound closure dynamics displayed by the indicated experimental groups. **D** RT-qPCRs measuring the abundance of p21 and p16INK4A transcripts in wounds from the indicated experimental groups (*n* = 3 each group) at day 6. **E** Schematic overview of the experimental procedures performed to assess the effects of p38 inhibition in doxorubicin-induced senescence in lungs. **F** p16 promoter-driven luciferase activities for the indicated experimental groups shown by p16-3MR mice 10 days after doxorubicin treatment. **G** RT-qPCRs of p21 and p16^Ink4a^ mRNAs in lungs from the indicated experimental groups. For p21 expression the number of animals is as follows: untreated (*n* = 3), Doxorubicin-treated (*n* = 6), and Doxorubicin-treated + SB203580 (*n* = 5). For p16Ink4 *n* = 3 of each group. Values are mean ± SD. *P < 0.05, **P < 0.01, ***P < 0.001 by two-tailed Student’s t test.

Finally, we tested the role of the p38 pathway in cutaneous wound healing and doxorubicin-induced cellular senescence. First, the rate of wound closure was measured in wild-type female mice treated daily with vehicle or with SB203580 and compared to that of Spry1^Y53A/Y53A^ mice (Fig. 7A). Topical treatment of wounds with SB203580 delayed wound healing to rates similar to those exhibited by Spry1^Y53A/+^ mice, which were in turn significantly lower than those of wild type mice (Fig. 7B, C). Expression analysis by qRT-PCR of wounds revealed a decrease of cellular senescence markers p21^Cip1^ and p16^Ink4a^ in the SB203580-treated group, comparable to that observed in Spry1^Y53A/+^ mice (Fig. 7D), indicating that cellular senescence of cells repopulating the wound was dependent on the activation of the p38 pathway. Likewise, bioluminescence of Spry1^+/+^; p16-3MR mice treated with a single dose of doxorubicin was reduced by chronic intraperitoneal administration of SB203580 (Fig. 7E, F), as it was expression of p21^Cip1^ in lungs (Fig. 7G). Again, expression of p16^Ink4a^ was not significantly reduced by SB203580, probably due to its modest induction in this experimental setting. Taken together, these data indicate that Spry-mediated activation of the p38 pathway is important for induction of cellular senescence in vivo.

## Discussion

In this work, we demonstrate a critical role of Sprouty proteins in induction and maintenance of cellular senescence in several in vitro and in vivo paradigms. We show that Sprouty1 and Sprouty2 are upregulated upon execution of cellular senescence, and that such induction is necessary and sufficient for implementation of a cellular senescence response both in vitro and in vivo. We also show that Sprouty proteins act upstream of the p38 pathways in these experimental paradigms.

Our previous work indicated that Sprouty1 is an important player in cellular senescence. First, expression of Spry1 in the thyroid carcinoma cell line TT induced a proliferative arrest with traits of cellular senescence both in vitro and in xenografts [26]. In these experiments, lentiviral-driven expression of Spry1 caused an increase in cellular senescence markers such as p16^ink4a^ and SAβ-Gal [26]. Second, genetic ablation of Spry1 caused a cell-autonomous increase in cellular proliferation of thyroid cells characterized by decreased SAβ-Gal staining and suboptimal expression/secretion of several SASP members [20]. Finally, in a recent report we show that heterozygous Spry1 mutant female mice presented persistent Wolffian ducts, consistent with failure of these epithelial structures to degenerated by programmed cellular senescence [7].

As stated above, the ERK1/2 pathway is considered to be the main target of Sprouty proteins. Our data do not support ERK1/2 inhibition as the mechanism by which Sprouty proteins induce cellular senescence. First, our experiments show chronic activation of the ERK1/2 pathway upon implementation of senescence by different triggers, despite strong induction of Spry1 and Spry2. Second, the ERK1/2 pathway was not inhibited by overexpression of Sprouty proteins in IMR90 cells. Third, no increase in ERK1/2 phosphorylation was seen in Spry1^Y53A/Y53A^ vs Spry1^+/+^ fibroblasts, either when growing in a 3T3 scheme or when acutely stimulated with different concentrations of FBS and for different time points. Accordingly, activation of the ERK1/2 pathway was not reduced by Spry1 overexpression in TT cells [26] or conversely, ERK1/2 phosphorylation was not increased in thyroid glands from Spry1 knockout mice in vivo [20]. We also found that deletion of Fgf2 via CRISPR/Cas9 did not prevent cellular senescence of cultured dermal fibroblasts. During development, the FGF receptors have been repeatedly shown to be a target of Spry proteins [37]. It is possible that functional redundance among FGFR ligands could mask their role in senescence of cultured dermal fibroblasts, or alternatively Sprouty proteins could be eliciting their effect on cellular senescence independently of FGFR signaling in this paradigm.

The observation that Sprouty proteins induce senescence is in agreement with a previous work showing that overexpression of Spry2 increases SA-β-galactosidase staining of BJ human diploid fibroblasts [38]. In that paper authors find that execution of a negative feedback loop silencing Ras-mediated signaling is responsible for induction of senescence caused by knockdown of NF1 or overexpression of oncogenic BRAF. We propose that Sprouty induction is the actual trigger of cellular senescence regardless of the resulting activation status of signaling pathways after implementation of negative feedback responses.

We show that the p38 pathway is activated upon exposure to senescence triggers, overexpression of Sprouty proteins, and culture of wild type murine fibroblasts over time. Such activation is not achieved when the Spry1 Y53A mutant is expressed in IMR90 fibroblasts (cultured in the absence of stressors or in the presence of etoposide), or in skin fibroblasts from Spry1^Y53A/Y53A^ mice. Our data also show that inhibition of the pathway using the drug SB203580 prevents cellular senescence both in vitro and in vivo. Activation of the p38 pathway is well known to be sufficient [39] and necessary for induction of cellular senescence in a wide variety of in vitro paradigms including replicative senescence [40], oncogenic Ras expression [41], DNA damage [42], or oxidative stress [43]. In vivo, muscle stem cells (satellite cells) become senescent with aging in a process that can be prevented by inhibition of the p38 pathway [44, 45].

We demonstrate that Tyrosine 53, along with the CIN85 and the Caveolin-1 binding sites are critical determinants of Sprouty’s ability to induce cellular senescence. The main interacting partner of the N-terminal tyrosine of Sprouty proteins is c-Cbl [1], an E3 ubiquitin-ligase that can also act as an non-proteolytic adaptor [46]. Interestingly, c-Cbl-CIN85 complex sets a scaffold that, together with GADD45, facilitates p38 activation by MEKK4 [47]. In support of this possibility, there are other studies pointing to a role of CIN85-c-Cbl complex in directly activating p38 signaling [48, 49]. On the other hand, interaction of Sprouty2 with protein phosphatase 2A, which dephosphorylates and inactivates p38 among many other substrates [50, 51], has been described [52, 53], although the physiological outcome of such interaction on p38 activity is unknown.

Another possibility is that Sprouty proteins regulate apical elements of the pathway such as generation of reactive oxygen species (ROS). Accumulation of ROS activate the p38 pathway not only in response to direct oxidants such as hydrogen peroxide but indirectly through activation of the ERK1/2 pathway in OIS [41, 54, 55] or upon DNA damage [56]. Moreover, oxidative stress caused by normal culture conditions (21% O_2_) plays a pivotal role in replicative senescence of human [57, 58] and specially of mouse cells [59]. Interestingly, Sprouty proteins contain a conserved cysteine-rich domain that coordinates an iron-sulfur cluster [60], thus opening the possibility that they respond to (and perhaps regulate) ROS levels. Accordingly, Sprouty proteins inhibit angiogenesis in vertebrates [61–63] and airway branching in Drosophila [64], suggesting that they sense and orchestrate adaptive responses to increased oxygen tension. In this regard, it has been shown that Sprouty2 downregulate HIF levels via proteasomal degradation [65], thus shutting down hypoxic responses and promoting oxidative phosphorylation with concomitants ROS production. Moreover, binding of Sprouty proteins to Caveolin-1 likely localize them to caveolae, which are cellular oxygen sensors [66]. All these considerations await future investigation.

## Materials and methods

### Mice

Mice were kept in a 12-h light/dark cycle, and food and water were supplied ad libitum. Spry1Y53A mice (Spry1^tm1.1Mns^) were produced in our laboratory as previously described [7]. Transgenic p16-3MR mice [Tg(Cdkn2a/luc/RFP/TK)1Cmps] were a generous gift of Dr. Judith Campisi (Buck Institute for Research on Aging, Novato, California), and were generated as described [31].

### Cell culture

The human diploid lung fibroblast cell line IMR90 (ATCC CCL186) and dermal fibroblasts were cultured in complete medium consisting on MEM supplemented with 2 mM L-Glutamine, 1% NEAA, 1% Sodium Pyruvate and 10% Heat-Inactivated Fetal Bovine Serum, all from Gibco. One percent (skin fibroblasts) or 0.5% (IMR90 cells) Penicillin-Streptomycin (Gibco) was also added to culture medium.

Dermal fibroblasts were extracted by removing the dorsal skin of newborn mice, which was incubated overnight at 4 °C floating on 0.25% trypsin (Gibco), with dermis facing down. Next day, the epidermis was removed and the dermis was digested with 0.5 mg/ml Type IV Collagenase for 30 min at 37 °C. Cells were then passed through a 70 µm cell strainer (Corning), and plated. For the 3T3 subcultivation protocol, 800,000 dermal fibroblasts were plated in 100 mm dishes and, after 3 days, counted with an hemocytometer and plated again at the same density.

To trigger cellular senescence of IMR90 fibroblasts, cells were incubated with 50 µM Etoposide (Selleckchem) during the whole length of treatments. Fresh medium was added every 3 days. For H_2_O_2_ treatments, cells were incubated with 600 µM H_2_O_2_ (Sigma-Aldrich) for 2 h, allowed to recover for 48 h and treated again for 2 additional hours. H_2_O_2_ was then removed and cells incubated with complete medium until the end of treatment. The p38 inhibitor SB203580 (Selleckchem) was added to cells at 10 µM final concentration. Medium was refreshed every 2–3 days.

### Lentiviral infection

Lentiviral constructs carrying wild type and mutant Sprouty genes or RasV12 were generated by PCR and cloned into a lentiviral plasmid (FUSPa) driving transgene expression from the Ubiquitin C promoter, and bearing a puromycin-resistance cassette. For genome editing, pLentiCRISPRv2 [67] was used. Lentiviruses were produced in 293T cell line transfected by the calcium phosphate method. Twenty-four hours after transfection, supernatants were collected, passed through a 0.45 µm syringe filter and directly added to target cells. Twenty-four hours after infection cells were selected with 2 µg/ml Puromycin (Sigma-Aldrich) for an additional 48–72 h.

### Western blotting

Protein extracts were obtained by lysing cells with a denaturing buffer containing 2% Sodium duodecyl sulfate (SDS) (Sigma-Aldrich) in 50 mM HEPES. After boiling and sonicating, protein extracts were subjected to immunoblot as described [68]. Antibodies used are detailed in Supplemental Table 1. For detection of Spry1 clones D9V6P and D9V6I were used indistinctly as both have proven specific using Spry1 knockout cells [[69] and data not shown].

### RT-qPCR

Total RNA was extracted with TRIZOL reagent (ThermoFisher), either by direct addition to tissue culture dishes or by homogenizing snap-frozen tissue using a TissueLyser LT device (Qiagen). RNA was reverse transcribed using the High-Capacity cDNA Reverse Transcription Kit (ThermoFisher) as per manufacturer’s instructions. Quantitative RT-PCR (qRT-PCR) reactions were performed by means of the SYBR green method, using either the 2x Master mix qPCR Low Rox kit (PCR Biosystems) or the Maxima SYBR Green qPCR Master Mix (ThermoFisher). The 2^-ΔΔCt^ method was used, normalizing to actin expression. Reverse transcriptase-minus and blank reactions were included in all experiments. Primers used are detailed in Supplemental Table 2.

### Cell proliferation assays and SA-β-Gal staining

For BrdU incorporation, cells were incubated with 4 µg/ml 5-bromo-2’-deoxyuridine for 24 h, fixed with 4% Paraformaldehyde and BrdU detected with an Anti-BrdU antibody (DAKO, cat# M0744). For clonogenic assays, cells were fixed with 4% Paraformaldehyde and stained with Crystal Violet (Sigma-Aldrich) for 30 min, washed 3 times with PBS and photographed. For SA-β-Gal staining, cells were fixed for 4 min with 0.5% Glutaraldehyde in PBS (pH 7.4) at room temperature. After 2 washes with PBS (pH 7.4), cells were rinsed with PBS pH 6.0 containing 1 mM MgCl_2_, and incubated with SA-β-galactosidase solution (2 mM MgCl_2_, 5 mM Potassium Ferricyanide, 5 mM Potassium Ferrocyanide and 1 mg/ml X-Gal in PBS pH 6.0) for 3 h to overnight at 37 °C. Next, cells were washed twice with PBS pH 7.4 and counterstained with 5 µg/ml Hoechst 33342.

### Wound healing assays

Wound healing assays were performed essentially as described [31]. Briefly, skin was wounded under Isoflurane anesthesia by using a 6 mm biopsy punch. For analgesia, Buprenorphine (0.05 mg/kg) was injected subcutaneously during the first 2 days after wounding. Every three days, pictures of wounds were taken and their area measured using ImageJ software. Topical treatments were administered diluted in acetone and applied directly to skin.

### Doxorubicin-induced senescence and bioluminescence imaging

We induced robust cellular senescence in vivo by following a Doxorubicin-based method previously described [32]. Briefly, a single dose of Doxorubicin (10 mg/kg) was injected intraperitoneally. Ten days later, senescence induction was confirmed in lungs by either RT-qPCR or whole-body luciferase activity. SB203580 or vehicle (PBS) were delivered intraperitoneally for the indicated times. Renilla luciferase activity was assessed by intraperitoneal injection of 15 μg/mice Xenolight RediJect Coelentarazine h (Perkin Elmer). Twenty-five minutes after injection, systemic luciferase activity was measured using a Photon Imager device (Biospace Lab).

### Transcriptomic analyses

Total RNA from dermal fibroblasts from three wild type and three Spry1^Y53A/Y53A^ mice was isolated at day 15 in vitro using RNAeasy columns (QIAGEN). RNA was reverse transcribed, and cDNA was hybridized to Affymetrix Mouse Gene 2.0 ST arrays, which contain >35,000 coding and non-coding transcripts as well as >2000 long intergenic non-coding transcripts. Enrichment analyses were conducted using the GSEA software [70, 71].

### Genome editing using CRISPR/Cas9

DNA oligonucleotides bearing sequences targeting mouse Fgf2 (GCCACTTCAAGGACCCCAAG) or loxP (GTATGCTATACGAAGTTATT) were hybridized and cloned into pLentiCRISPRv2, a gift from Feng Zhang (Addgene plasmid # 52961; http://n2t.net/addgene:52961; RRID:Addgene_52961). Cells were infected the day after establishment of the culture and selected with 2 µg/ml puromycin 2 days later, for 2–3 additional days. To assess editing efficiency, genomic DNA was extracted and amplified with primers 5’-TTGTACACTCAAGGGGCTCTC-3’ and 5’-CCGCCGTTCTTGCAGTAGA-3’ targeting the first exon of mouse Fgf2 gene. PCR products were subjected to Amplicon sequencing (Genewiz), and analyzed using CRISPResso2 [72].

### Statistics and reproducibility

The data are presented as the means with standard deviation (± s.d.) derived from a minimum of *n* = 3 independent experiments. Significance was assessed using a two-tailed Student’s t-test (*P < 0.05, **P < 0.01, ***P < 0.001) across all experiments. Statistical analyses were conducted using Prism 9, with the exception of generating GSEA plots, which were created using the GSEA platform. Sample sizes were not predetermined using statistical methods; however, they align closely with those reported in prior studies in the field [33, 34]. Normality of data distribution was assumed but not formally tested. Data collection and analysis were performed under blind conditions whenever feasible, and no data points were excluded from the displayed graphs. Animal treatments were randomly delivered.

### Supplementary information

Supplemental Figure 1

Supplemental Figure 2

Supplemental Figure 3

Supplemental Table 1

Supplemental Table 2

Uncropped blots

## Data Availability

All datasets are presented in the main manuscript or additional supporting files.
