# Supplementary material for: Sprouty1 is a broad mediator of cellular senescence
Source: Cell Death Dis. 2024 Apr 26;15(4):296. doi: 10.1038/s41419-024-06689-4 (PMC11053034; doi:10.1038/s41419-024-06689-4)
Supplement: Supplementary file 1 — Supplemental Figure 1 [file 41419_2024_6689_MOESM1_ESM.pdf]

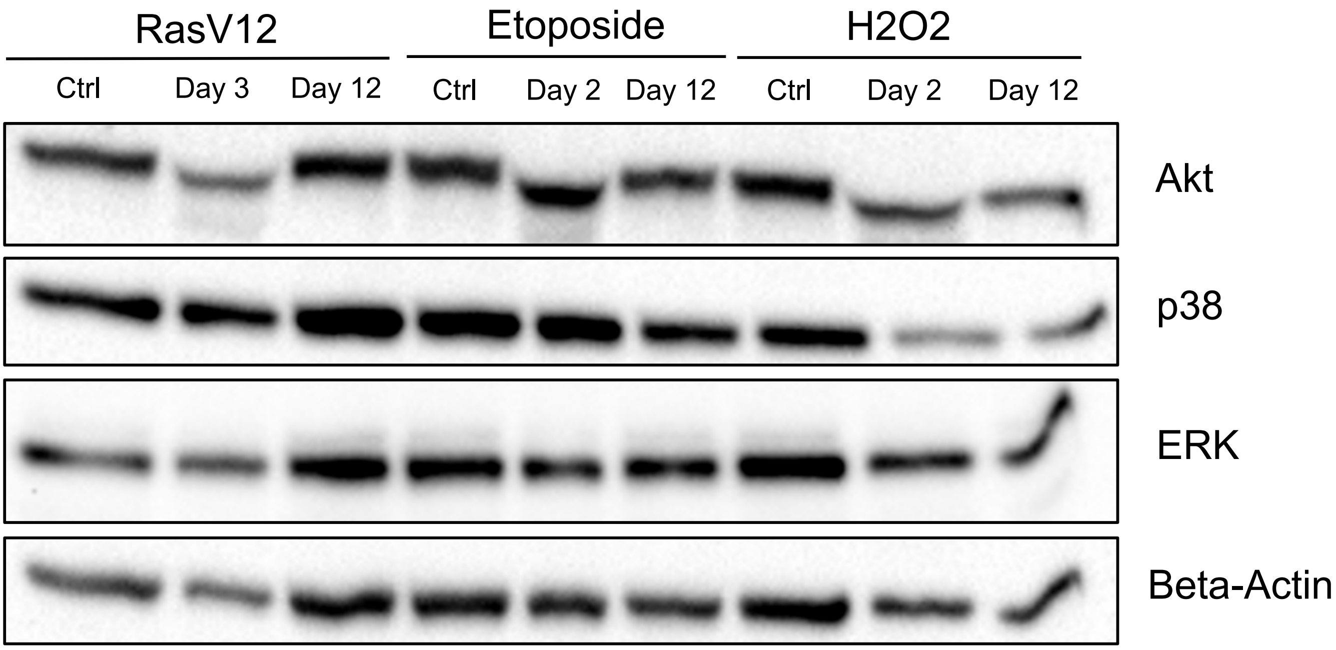

Supplemental Figure 1. Expression of RasV12, or treatment with Etoposide or H2O2 do not alter steady state levels of Akt, p38 or ERK in imr90 fibroblasts. Cells were treated as above and lysates probed with the indicated antibodies to Akt (Santa Cruz #sc-1618), p38 (Cell Signaling #1679212) or ERK (BD Transduction #610124).
