# Supplementary material for: Sprouty1 is a broad mediator of cellular senescence
Source: Cell Death Dis. 2024 Apr 26;15(4):296. doi: 10.1038/s41419-024-06689-4 (PMC11053034; doi:10.1038/s41419-024-06689-4)
Supplement: Supplementary file 2 — Supplemental Figure 2 [file 41419_2024_6689_MOESM2_ESM.pdf]

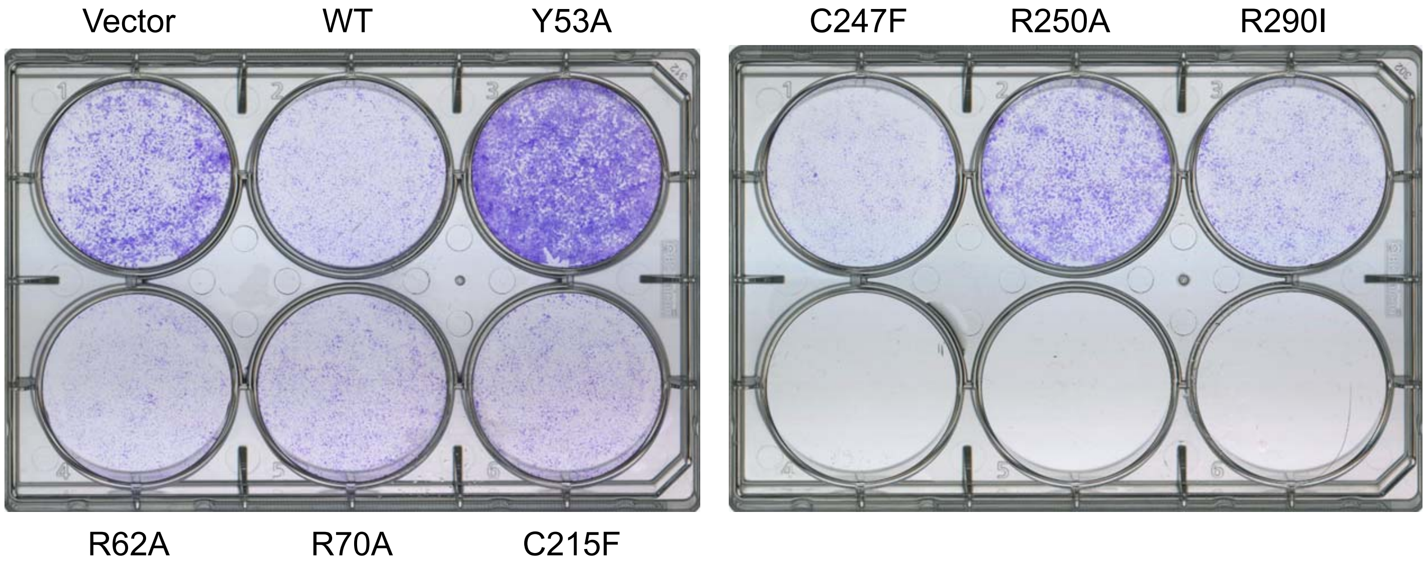

Supplemental Figure 2. Clonogenic assays of IMR90 fibroblasts infected with the indicated constructs encoding Spry1 mutants. The conserved N-terminal tyrosine in Spry1 is Y53. Mutations R62A and R70A correspond to binding sites of CIN85, whereas R250A eliminates the Caveolin-1 binding site. Mutations C215F,C247F and R290I have been described in some cancer patients
