# Supplementary material for: Sprouty1 is a broad mediator of cellular senescence
Source: Cell Death Dis. 2024 Apr 26;15(4):296. doi: 10.1038/s41419-024-06689-4 (PMC11053034; doi:10.1038/s41419-024-06689-4)
Supplement: Supplementary file 3 — Supplemental Figure 3 [file 41419_2024_6689_MOESM3_ESM.pdf]

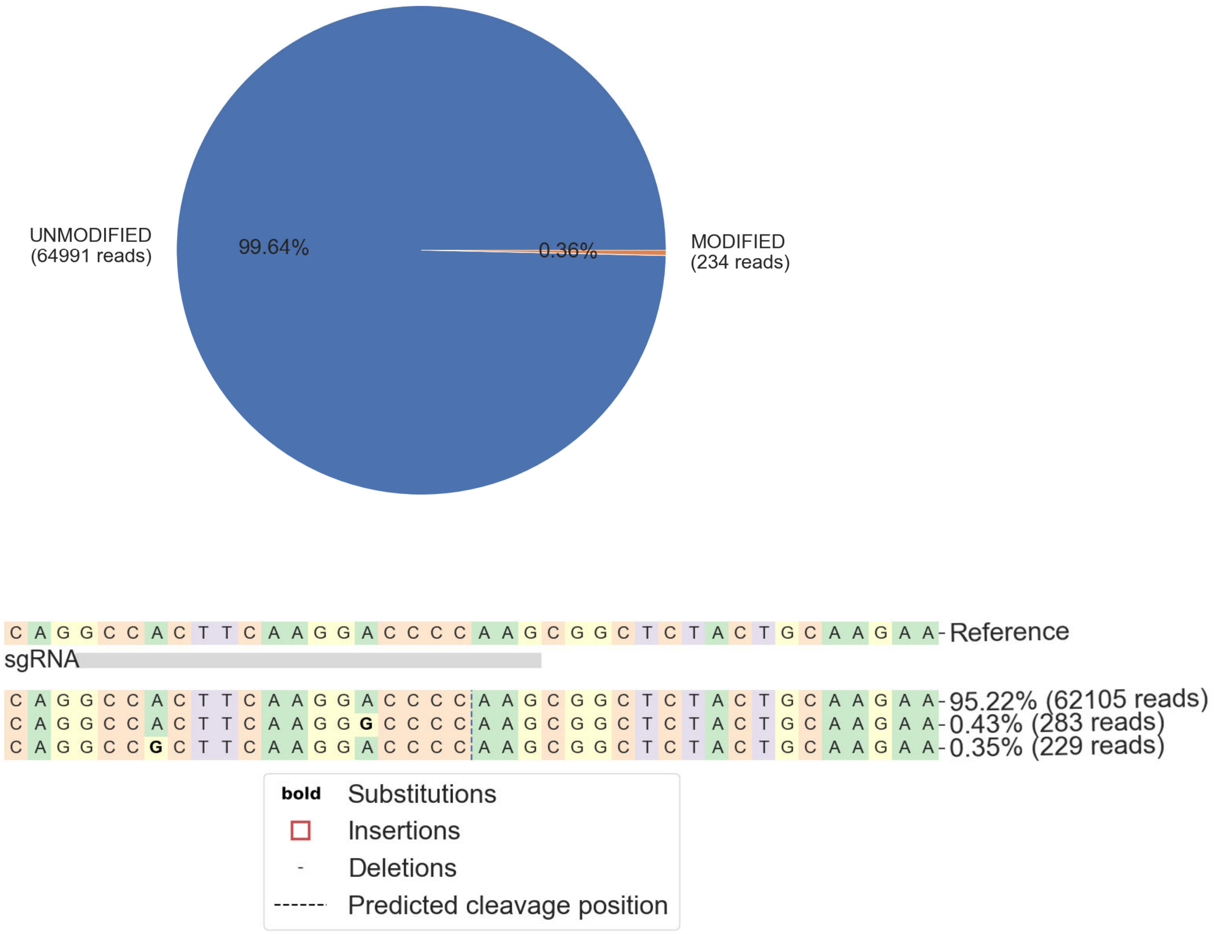

Supplemental Figure 3. Top panel, percentage of sequence modifications generated by the loxP sgRNA around the cleaving site of the Fgf2 sgRNA in skin fibroblasts. Bottom panel, sequence modifications and their read counts. Note that unlike indels induced by the sgRNA targeting Fgf2, changes detected here are substitutions, probably constituting base call errors during sequencing.
