# Supplementary material for: Sprouty1 is a broad mediator of cellular senescence
Source: Cell Death Dis. 2024 Apr 26;15(4):296. doi: 10.1038/s41419-024-06689-4 (PMC11053034; doi:10.1038/s41419-024-06689-4)

Figure 1A

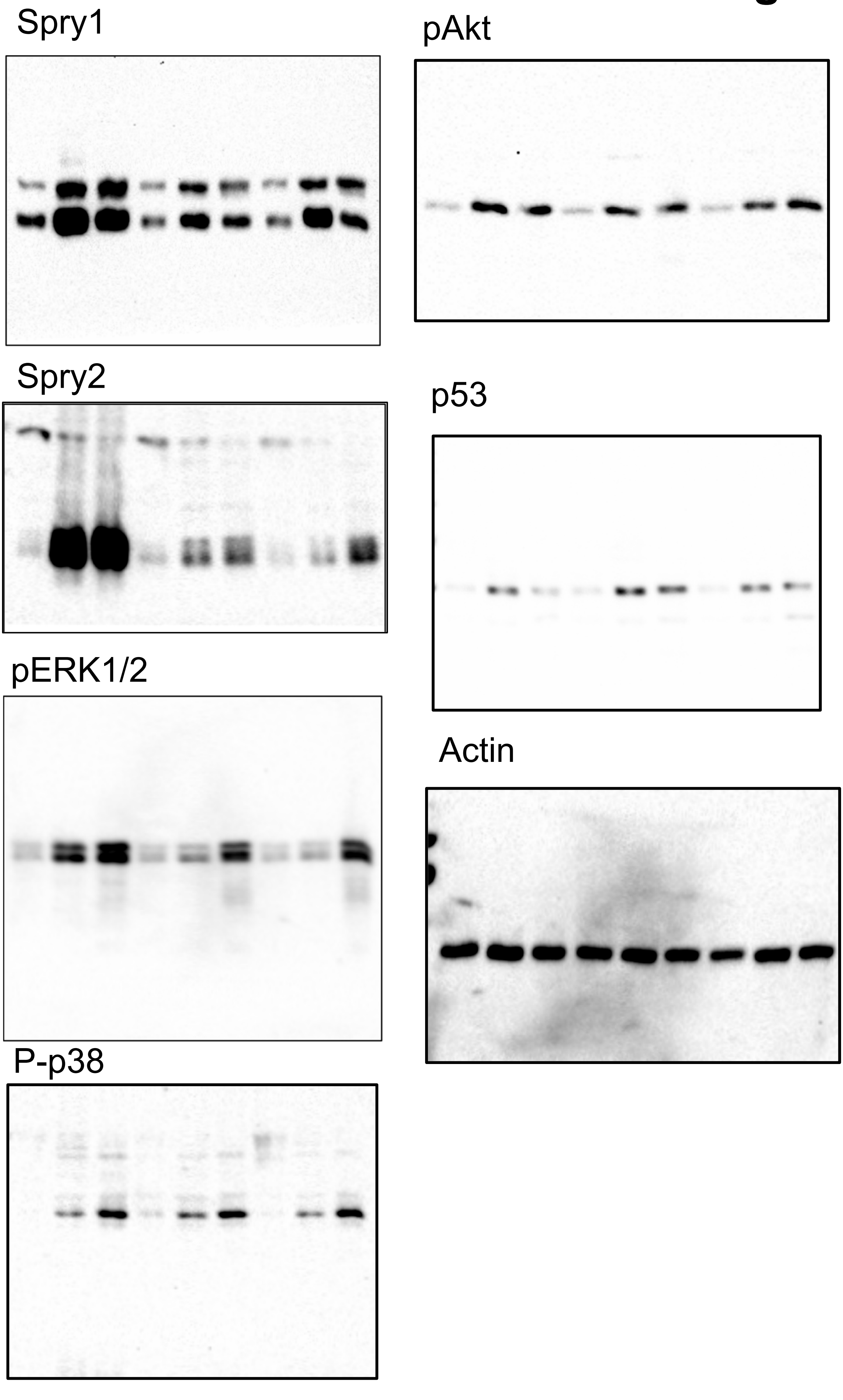

Figure 2B

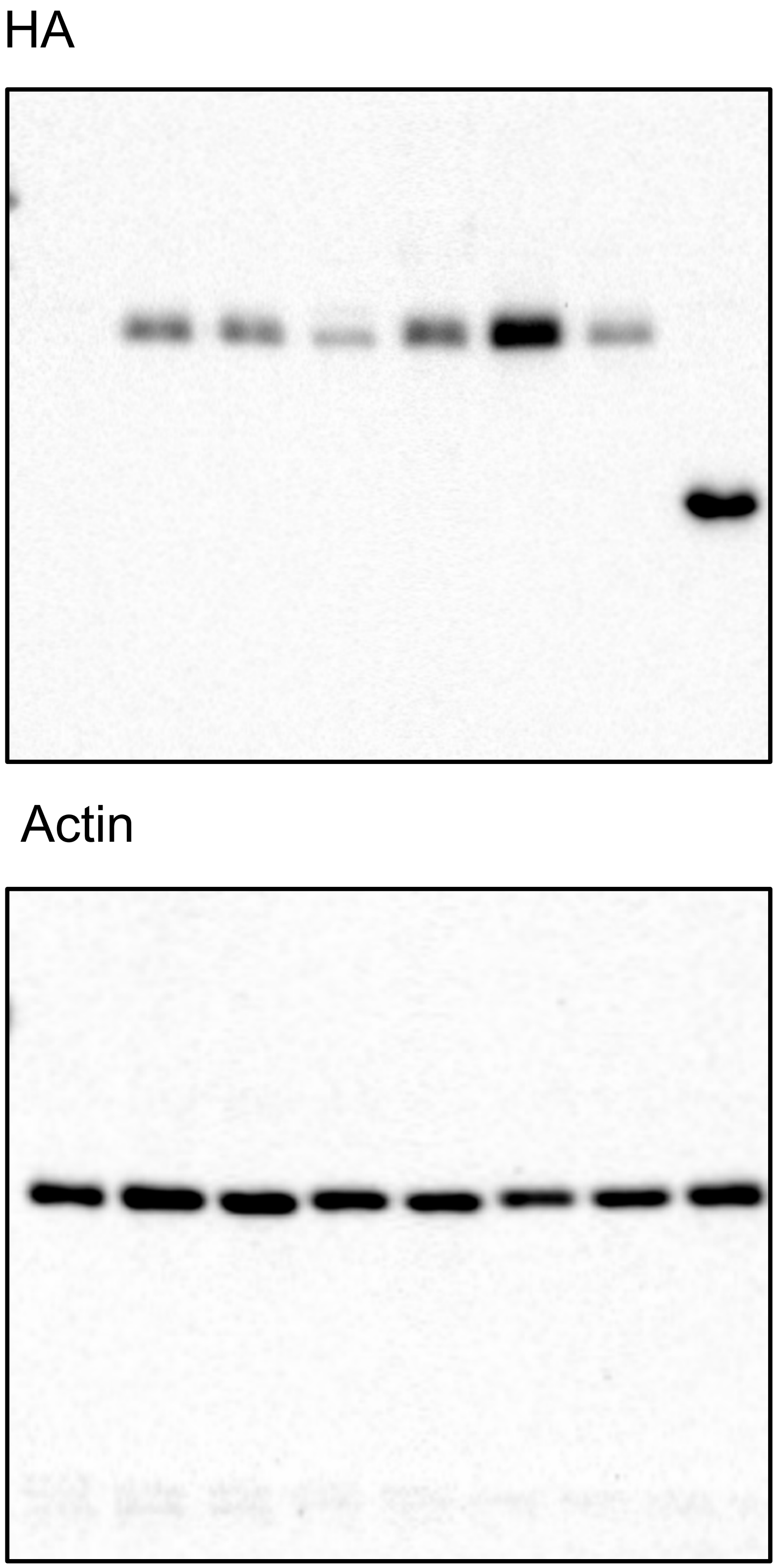

Figure 3D

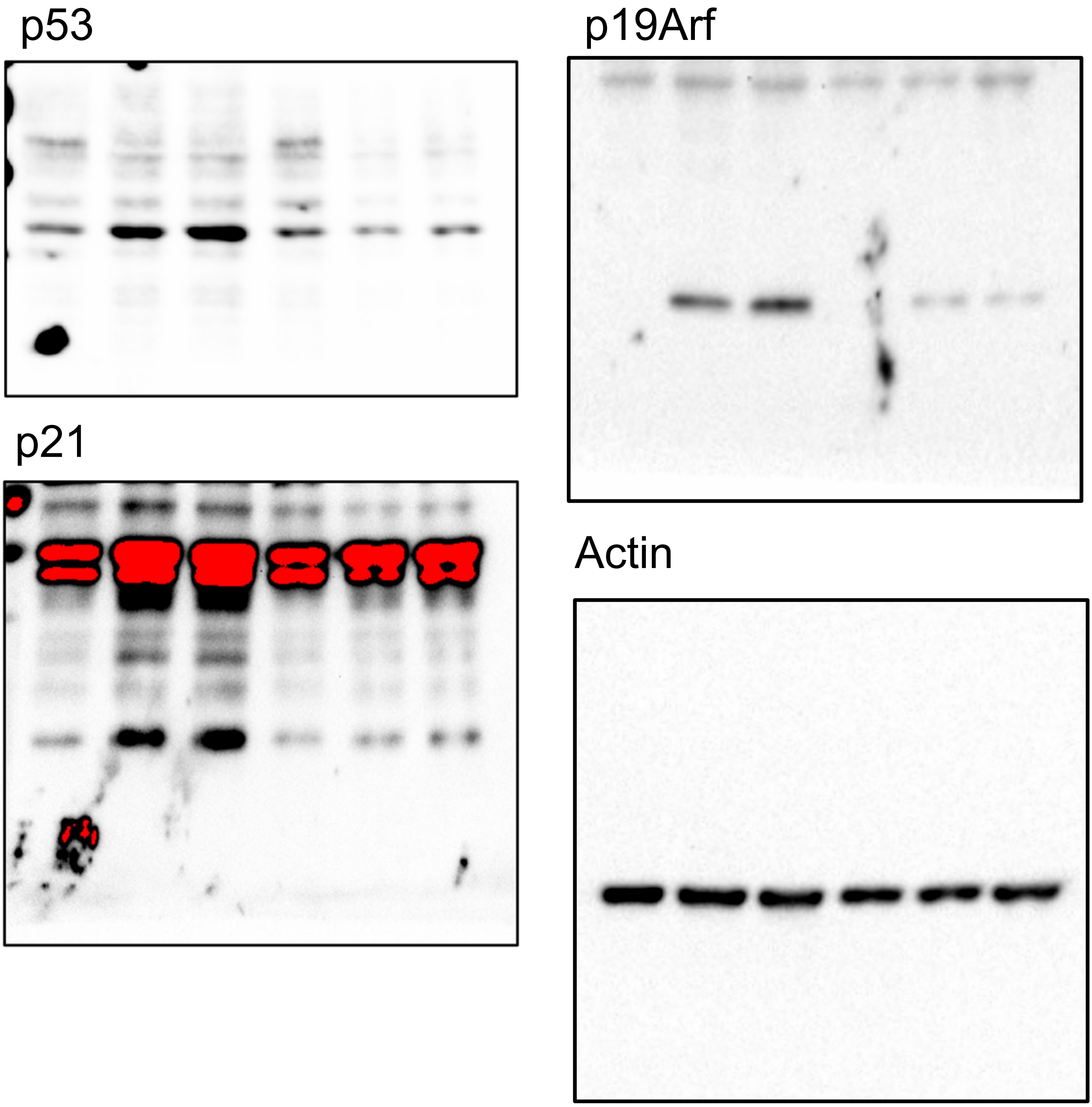

Figure 5A

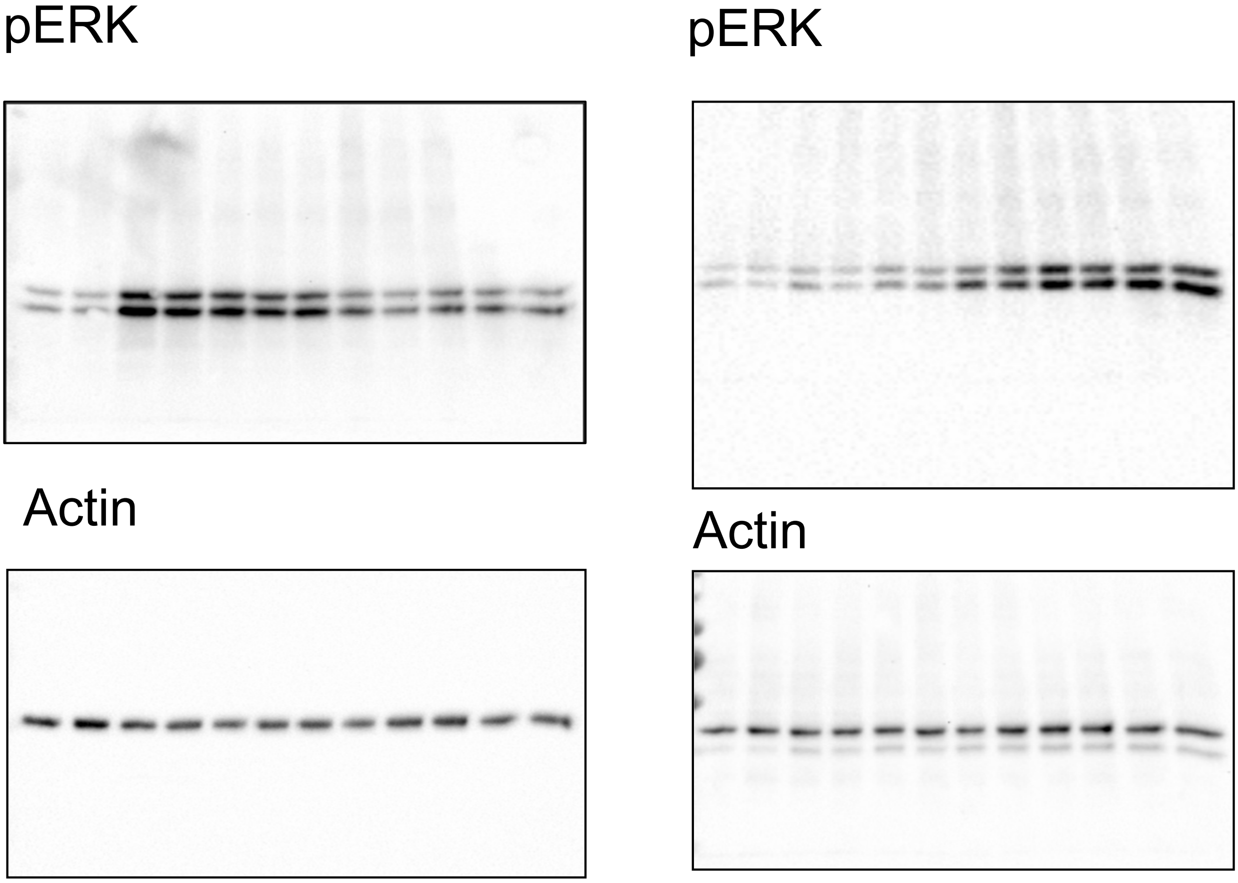

Figure 6A

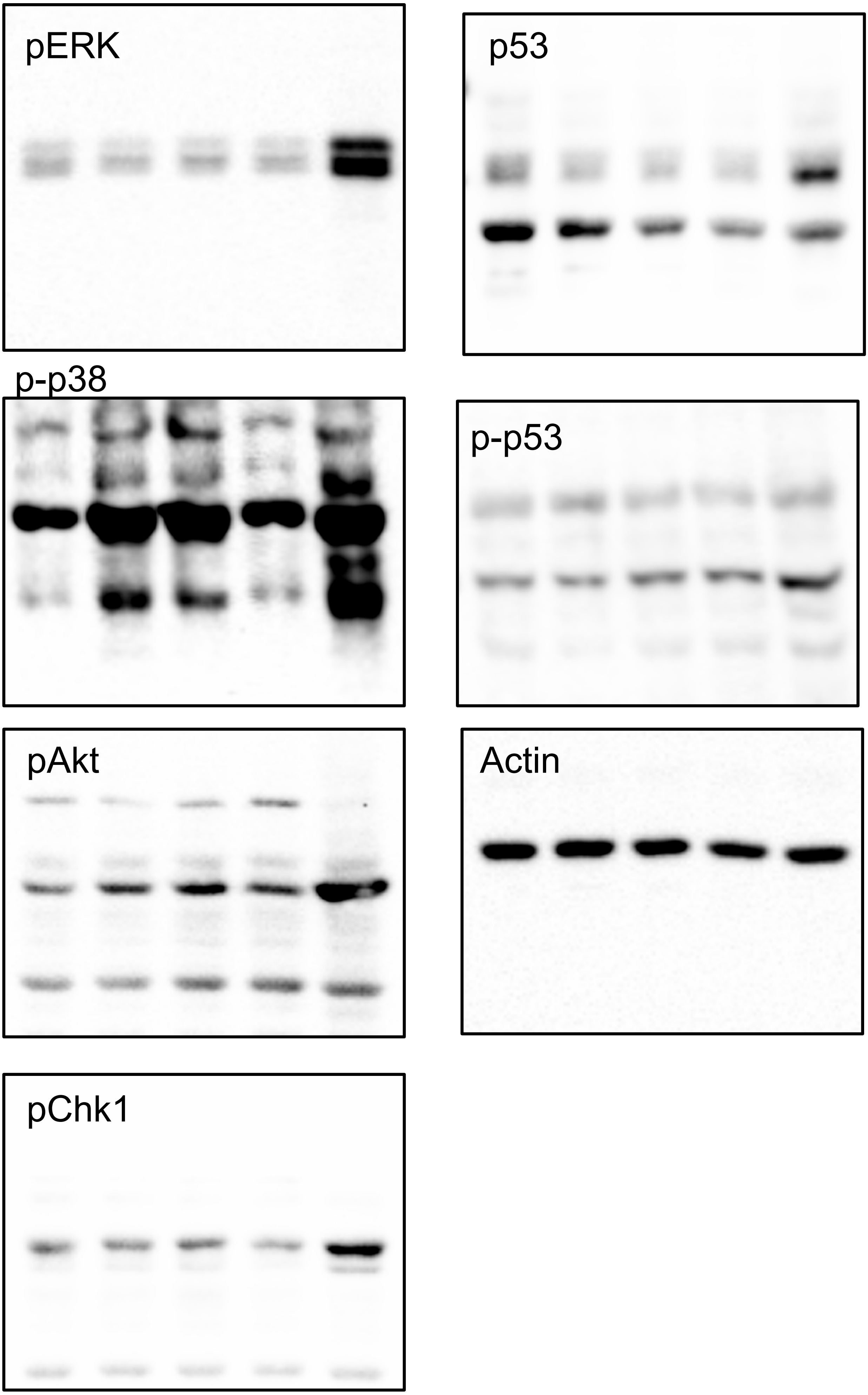

Figure 6B

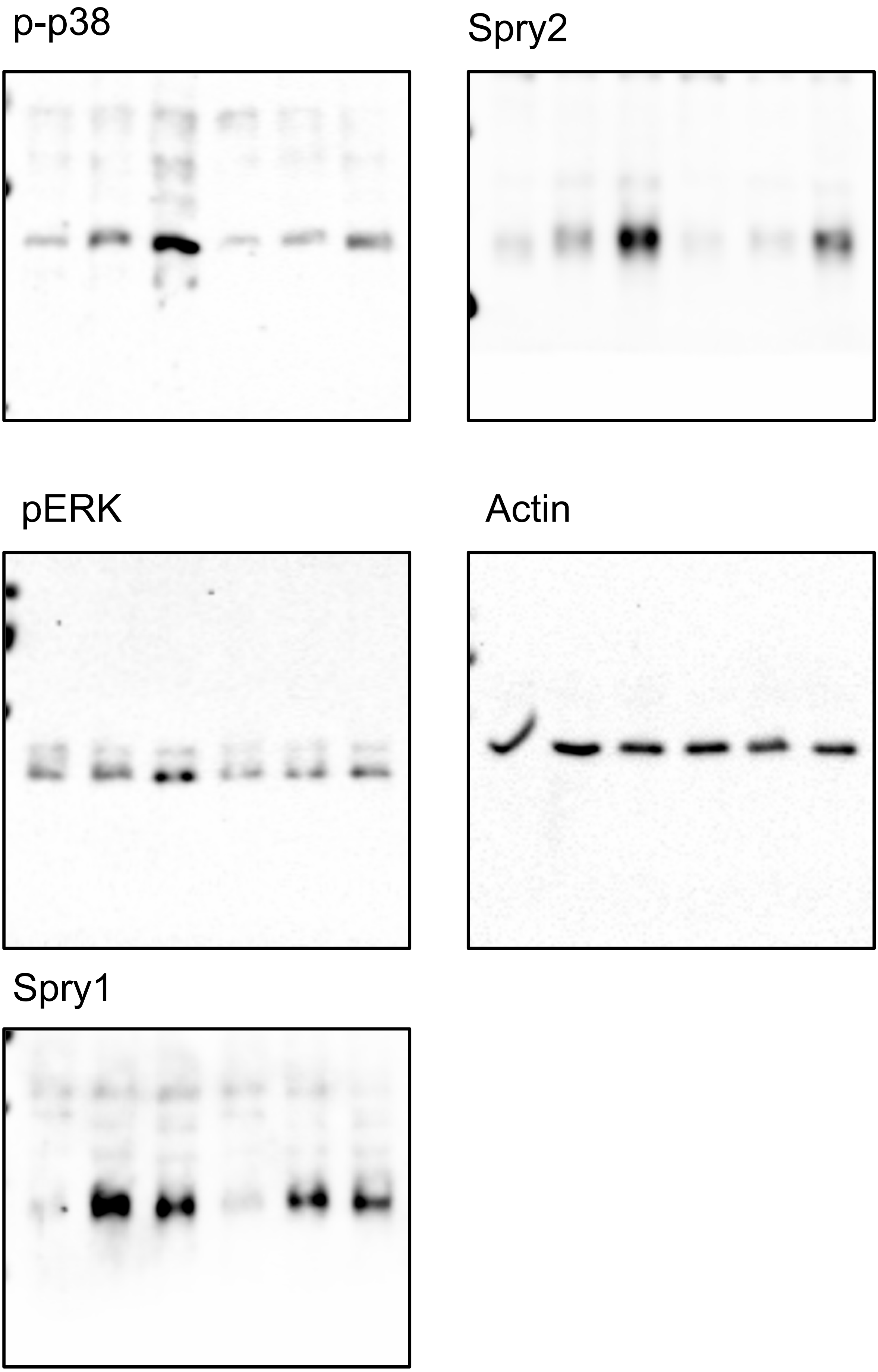

Figure 6C

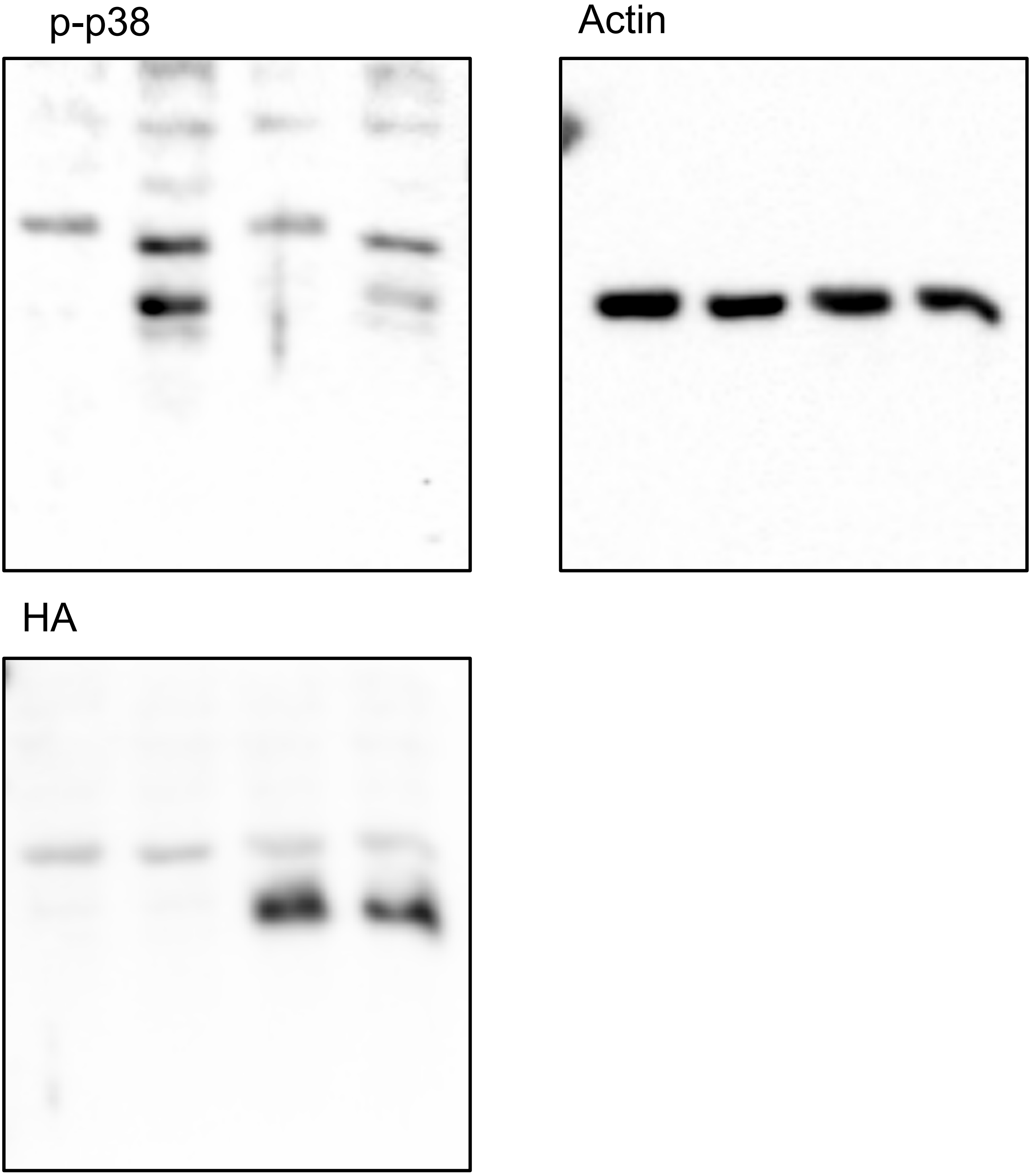

Figure 6H

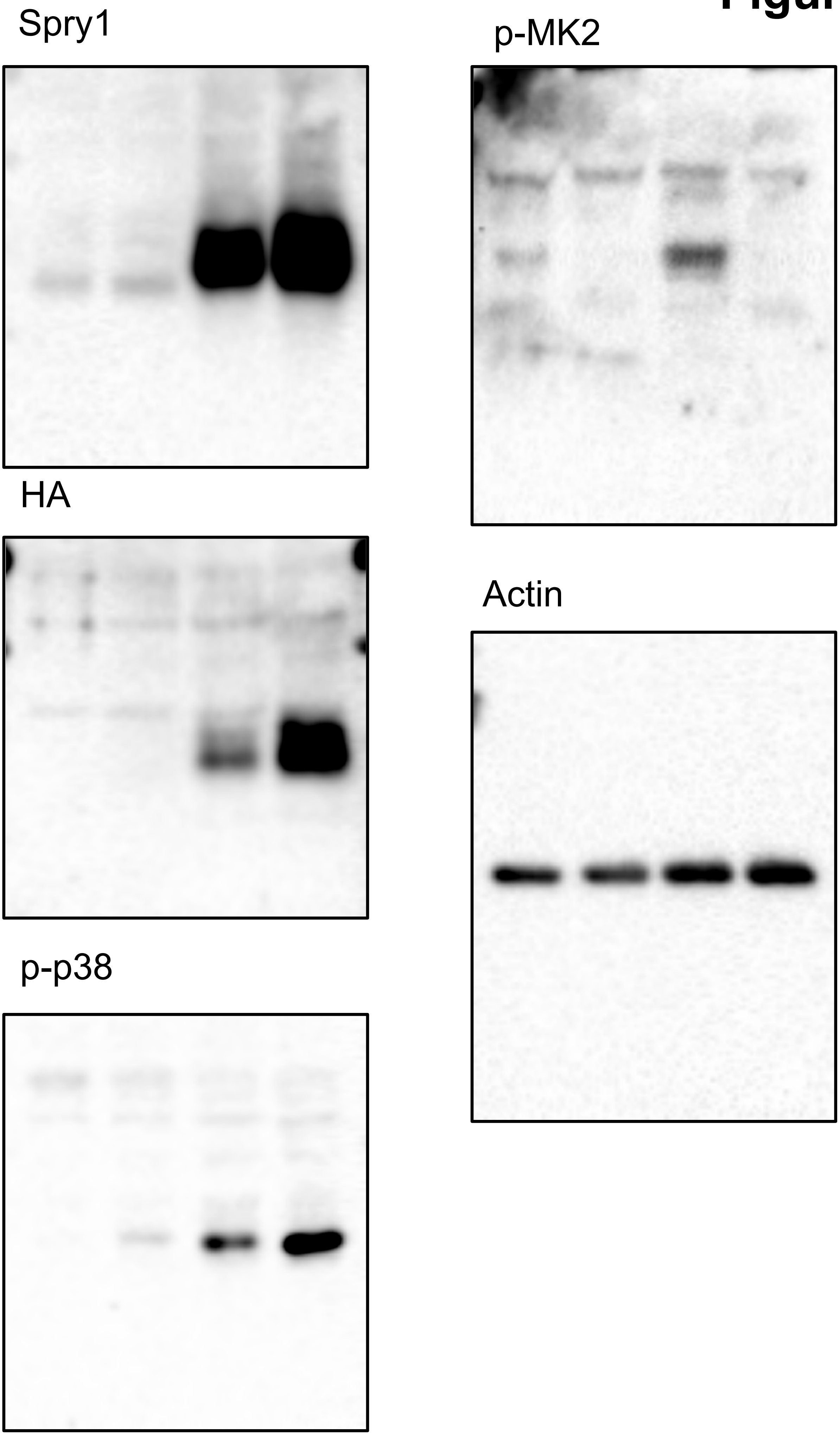

Supplemental Figure 1

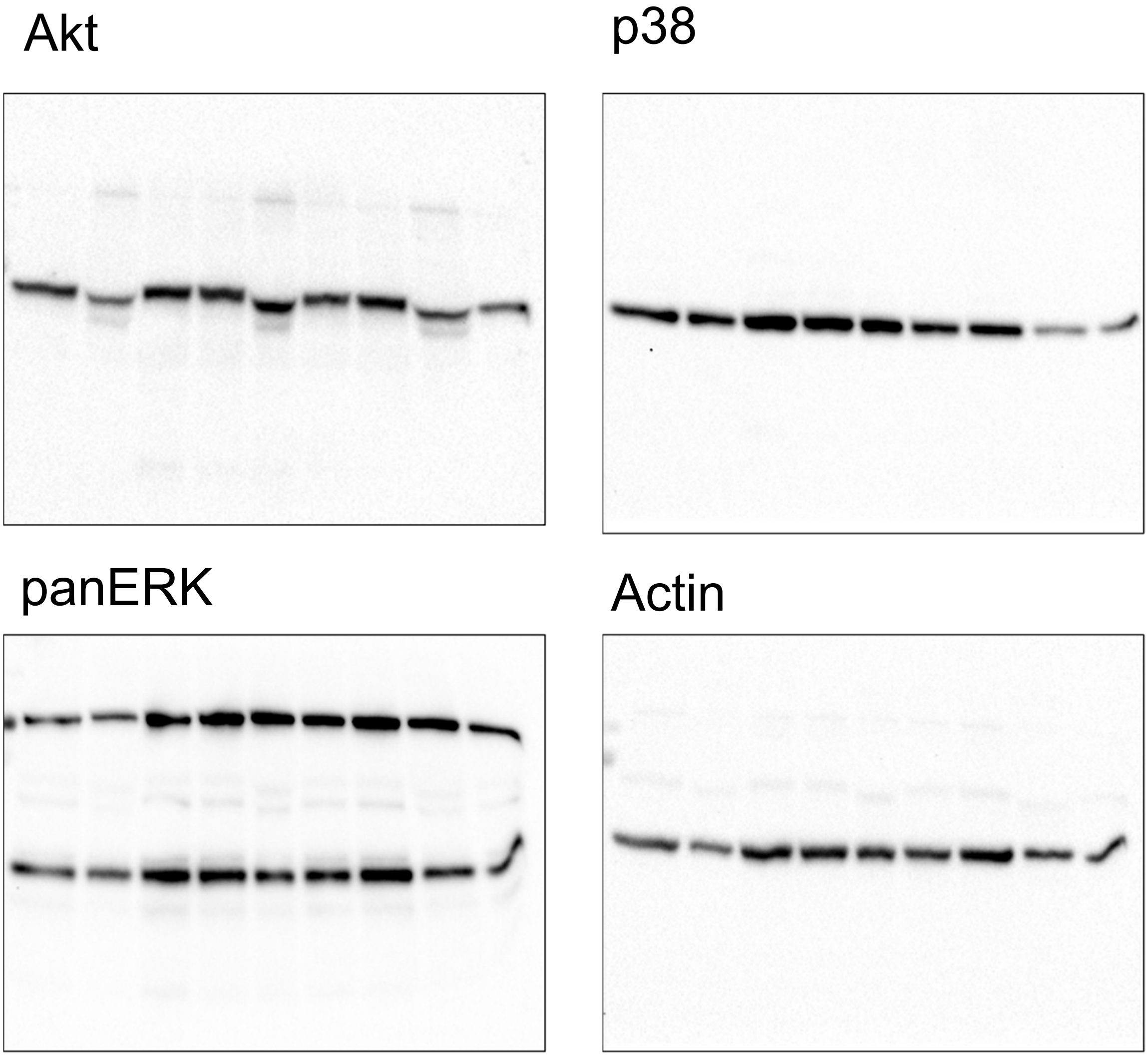

Supplement: Supplementary file 6 — Uncropped blots [file 41419_2024_6689_MOESM6_ESM.pdf]
